# Barriers and Opportunities to Include Underrepresented Population Groups in Vaccine Trials: Cross-Sectional, Observational, Online Survey Study From the VACCELERATE Research Network

**DOI:** 10.2196/89025

**Published:** 2026-04-07

**Authors:** Mine Durusu Tanriover, Dimitrios Poulimeneas, Grammatiki-Christina Tsopela, Ioannis Kopsidas, Christos Argyropoulos, Romina Di Marzo, Olena Valdenmaiier, Stine Finne Jakobsen, Paula Valle Simon, Antonio Javier Carcas-Sansuan, Ole F Olesen, Oliver A Cornely, Zoi Dorothea Pana, Theoklis Zaoutis, Murat Akova

**Affiliations:** 1 Department of Internal Medicine, Faculty of Medicine, Hacettepe Üniversitesi Aşı Enstitüsü Sıhhiye, Ankara, 06230, Turkey, 90 312 305 3069; 2 Vaccine Institute, Hacettepe University, Ankara, Turkey; 3 Collaborative Center for Clinical Epidemiology and Outcomes Research (CLEO), Athens, Greece; 4 Chemical Engineering Department, King Fahd University of Petroleum & Minerals (KFUPM), Dhahran, Saudi Arabia; 5 European Vaccine Initiative (EVI), Heidelberg, Germany; 6 Centre of Excellence for Health, Immunity and Infections, Rigshospitalet, University of Copenhagen, Copenhagen, Denmark; 7 Hospital La Paz Institute for Health Research (IdiPAZ), Madrid, Spain; 8 Servicio Madrileño de Salud, Madrid, Spain; 9 Cologne Excellence Cluster on Cellular Stress Responses in Aging-Associated Diseases (CECAD), Institute of Translational Research, Faculty of Medicine and University Hospital Cologne, University of Cologne, Cologne, Germany; 10 Department of Internal Medicine, Center for Integrated Oncology Aachen Bonn Cologne Duesseldorf (CIO ABCD) and Excellence Center for Medical Mycology (ECMM), Faculty of Medicine and University Hospital Cologne, University of Cologne, Cologne, Germany; 11 Clinical Trials Centre Cologne (ZKS Köln), Faculty of Medicine and University Hospital Cologne, University of Cologne, Cologne, Germany; 12 Center for Molecular Medicine Cologne (CMMC), Faculty of Medicine and University Hospital Cologne, University of Cologne, Cologne, Germany; 13 School of Medicine, Department of Basic & Clinical Sciences, University of Nicosia, Nicosia, Cyprus; 14 Department of Infectious Diseases and Clinical Microbiology, Faculty of Medicine, Hacettepe University, Ankara, Turkey

**Keywords:** barriers, COVID-19, vaccinations, pandemic preparedness, vaccine trials, volunteer registry

## Abstract

**Background:**

Despite the vast growth of vaccine studies during the SARS-CoV-2 pandemic, clinical trials failed to adequately represent diverse societal groups, resulting in the underrepresentation of specific populations. Understanding the factors hampering participation in vaccine clinical trials is essential to better identify structural, ethical, and communication barriers and to improve inclusive strategies for broader and more equitable participation in future vaccine research.

**Objective:**

This study aimed to identify the perceived barriers to participation in vaccine trials among pregnant and lactating women, children aged younger than 18 years, and adults aged older than 65 years, as reported by professionals with expertise in vaccines or vaccine trials.

**Methods:**

An online questionnaire was developed to gather personal information, group-specific barriers to vaccine trial participation, and suggestions to overcome these barriers. Data are presented as absolute (n/N) and relative frequencies (%).

**Results:**

A total of 115 respondents, the majority (n=73, 63.5%) of whom were working in the scientific community, completed the online survey. Challenges in recruiting children were identified due to “safety or efficacy concerns,” “difficulties about ethics and regulatory issues,” and “lack of targeted information and communication.” Challenges in recruiting pregnant and lactating women were primarily “ethics and regulatory requirements,” “safety issues,” and “lack of prioritization or interest.” “Lack of information and communication channels adapted to the specific target group,” along with “lack of prioritization,” were the main challenges in recruiting older participants. Provision of health-related incentives, including but not limited to access to new treatments and receiving expert medical care, seems to be the top-rated motivation to participate in vaccine clinical trials.

**Conclusions:**

The main challenges in recruiting pregnant and lactating women and children in vaccine trials involve safety and efficacy concerns, as well as lengthy ethical and regulatory processes. For older adults, key issues include poor communication channels tailored to their needs, limited information, lack of prioritization, funding, infrastructure, and industry interest. Across all underrepresented groups, low awareness of and poor communication about research opportunities were major barriers. Additionally, mobility issues affected older adults, while lack of motivation and incentives affected children, and low health literacy and provider uncertainty impacted pregnant and lactating women. Improving communication infrastructure and enhancing communication strategies with clear, tailored messages to build trust and motivate participation are essential to improve inclusion in vaccine research.

## Introduction

Ensuring a diverse representation of population groups in clinical trials is essential for advancing health equity, as these groups may have different risk factors and variable responses to medical products [1]. Despite the rapid expansion of vaccine studies during the SARS-CoV-2 pandemic, clinical trials failed to adequately represent diverse societal groups, resulting in the underrepresentation of specific populations [2]. Underrepresented groups could include people who have inadequate access to health care for any reason, including unreachable or inadequate vaccination systems, health care provider discrimination, lack of health care provider recommendations, or legal restrictions [3,4]. A cross-sectional study examining trials registered at ClinicalTrials.gov identified that among 230 vaccine trials based in the United States, Black or African American individuals (constituting 10.6% of all participants) and adults aged 65 years or older (constituting 12.1% of the participants) were underrepresented compared with US census data [5]. On the other hand, certain populations with access to health care may still be underrepresented in clinical trials largely because of the exclusion of these population groups right at the initial stage of vaccine trial design. For instance, only 8 (9.5%) clinical trial protocols among the 84 phase II and phase III vaccine clinical trials reporting from 2009 to 2019 explicitly included pregnant people [6]. A semistructured qualitative interview conducted in the United Kingdom showed that although pregnant women acknowledged the importance of vaccine trials to demonstrate the safety and effectiveness of vaccines, they were reluctant about their own involvement in such trials [7].

We cannot underestimate the impact of increasing vaccine hesitancy on the underrepresentation of certain populations in vaccine clinical trials. For instance, Willis et al [8], who surveyed 1205 Arkansas adults in July and August of 2020 to evaluate COVID-19 vaccine hesitancy, showed that among Black or African American populations, 50% of respondents showed hesitancy toward receiving the COVID-19 vaccine, compared to 18.37% of White respondents.

Recently, Daniel et al [9] investigated the enablers and barriers to participation in vaccine trials. Safety concerns, institutional distrust and misinformation, sociocultural and religious norms, and logistical burdens were the most prominent barriers overall. Safety concerns were the predominant barrier among pregnant and pediatric participants. Recruiting children can be particularly challenging and may even lead to early trial termination due to failure to meet recruitment targets [10].

Strategies to better ensure diverse representation in vaccine clinical trials, particularly in preventive vaccine studies, are needed. Understanding the factors hampering participation in vaccine clinical trials for certain population groups may enhance future access to and engagement with a diverse selection of volunteers in clinical trials and facilitate better promotion of tools such as the VACCELERATE Volunteer Registry [11-17]. In addition to pinpointing the most underrepresented population groups, it is also crucial to identify the barriers that prevent professionals from involving these population groups in vaccine trials.

Within the VACCELERATE consortium, a task force was assigned to launch, disseminate, and analyze an extended online survey targeted at professionals with expertise in vaccines or vaccinations, with the aim of identifying barriers to participation in vaccine trials among pregnant and lactating women, children (aged <18 y), and older adults (aged >65 y) in the European region. For this reason, an internal consultation addressed to the National Coordinators was conducted to identify the challenges of vaccine trial participation of the underrepresented population groups [11]. Although the underrepresented populations could be diverse, based on the results of the initial survey, as well as a consultation with experts from the European Centre for Disease Prevention and Control and the World Health Organization, the task force selected (1) children (aged <18 y), (2) pregnant and lactating women, and (3) older adults (aged >65 y) to be the primary focus of the extended survey. Hence, this study focuses specifically on children, pregnant and lactating women, and older adults for the purposes of the analysis.

This study aimed to identify the perceived barriers to participation in vaccine trials among pregnant and lactating women, children aged younger than 18 years, and adults aged older than 65 years, as reported by professionals with expertise in vaccines or vaccine trials.

## Methods

### Study Design and Population

VACCELERATE is a pan-European clinical research network established to accelerate vaccine trials and pandemic preparedness [18]. The project aimed to streamline and harmonize clinical trial efforts across Europe. One of the work packages was dedicated to designing and implementing a European Union–wide, dynamic, harmonized, and sustainable volunteer registry for phases II and III vaccine clinical trials with an initial focus on the COVID-19 pandemic and for the future expansion to any forthcoming European epidemic or pandemic. Within this work package, the study was designed to identify the causes of underrepresentation of pregnant and lactating women, children aged younger than 18 years, and adults aged older than 65 years as perceived by professionals in the vaccine field. It was not intended to explore the reasons why these population groups are not targeted at the trial design stage or to compare the magnitude of differences and the impact of perceived barriers across the underrepresented groups. Indeed, we did not inquire on the reasons why most of the vaccine trials were excluding these population groups and how we could get sponsors to design trials that are more inclusive and diverse. Instead, we have assumed that trials are open to children, pregnant and lactating women, and older adults.

An online 11-item questionnaire, divided into 3 sections, was developed by the authors. The full questionnaire can be found in Multimedia Appendix 1. The authors were engaged in several rounds of discussion to list the most appropriate response options for each group, as analyzing the results of the open-ended questions in this heterogeneous group of underrepresented populations would have been particularly challenging. Section A consisted of 6 questions, aiming to capture personal information of the survey respondents, such as (1) the capacity in which respondents filled out the survey (expert opinion or organization representative), (2) the underrepresented population groups that the respondents had experience with (children, pregnant and lactating women, and/or older adults), (3) affiliation of the respondent (civil society, funder, industry, and public health agencies), (4) geographical area of expertise, (5) highest level of expertise (spanning from junior to executive), and (6) total years of experience in vaccines and vaccination. Section B aimed to capture group-specific barriers to vaccine trial participation of certain population groups. The questions of section B followed a smart-branching design based on the answers of the respondents in section A. More specifically, in questions 7, 8, and 9 (referring to children, pregnant and lactating women, and older adults, respectively), survey respondents were asked to agree or disagree with statements provided regarding (1) the reasons they are unable to recruit volunteers from the given population group and (2) the main reasons the given population group does not participate in clinical trials as the professionals perceive from their side. Section C was titled “Overcoming Barriers” and involved 2 questions. Question 10 was a free text question, asking professionals for their opinion on what could improve equal access and representation of underrepresented population groups in COVID-19 vaccine research. Finally, question 11 asked on motivators of individuals to participate in a volunteer registry.

The VACCELERATE consortium included National Coordinators from Austria, Belgium, Cyprus, the Czech Republic, Denmark, France, Germany, Greece, Hungary, Ireland, Italy, Lithuania, the Netherlands, Poland, Portugal, Serbia, Slovakia, Spain, Sweden, Israel, Norway, Switzerland, and Turkey. A pilot study was done with the National Coordinators to ensure that the online platform for the survey was working appropriately. As these coordinators were all experts in vaccine research from 23 different countries, this pilot study also ensured that the questions were valid and sound before the survey questionnaire was circulated more widely. No patient or trial participant was targeted either in the pilot phase or in the actual survey.

To reach as many professionals as possible, National Coordinators were asked to list relevant agencies and organizations (ie, public health authorities, academia, nongovernmental organizations, and research development departments) in their network. After mapping down the potential participants, we created an extensive list of possibly eligible professionals. We used this list to disseminate our survey and attract more professionals with opportunity sampling, given that effective communication and collaboration with professionals are key to survey success. Invitation letters (Multimedia Appendix 2) included a statement to point out that the survey targeted professionals with expertise in vaccines or vaccinations to enroll qualified respondents.

### Ethical Considerations

#### Ethics Approval

The study was conducted in accordance with the Declaration of Helsinki and approved by the Hacettepe University Ethics Committee (approval 2023/06‐30; approved on April 4, 2023).

#### Informed Consent

Informed consent was obtained from all survey respondents prior to answering the questions of the survey. Respondents could withdraw at any point before submission by simply closing their web browser.

#### Privacy and Confidentiality

The survey was hosted by LimeSurvey, a platform fully compliant with the General Data Protection Regulation, and was accessible via the VACCELERATE stakeholder survey link [19]. All collected data were anonymized; no traceable information was collected.

After clicking on submission, there was no way of identifying the respondent or associating their answers with them. As such, the answers could not be revoked once submitted. No identifying information of the participants was collected or published.

#### Participant Compensation

Participants did not receive any compensation for participation.

### Statistical Analysis

Data are presented as absolute (n/N) and relative frequencies (%). Statistical analysis was performed using Microsoft Excel. Free-text data are presented as raw answers and categorized.

## Results

### Overview

As a first step, a list was prepared, including contact information of professionals and institutions to serve as the basis of the dissemination process. Afterward, the list was refined to contain 219 email addresses from 26 countries. Invitation letters for the professionals to participate in the online survey were initially sent on June 8, 2023. The invitation letter and the link to the survey were also distributed through personal communication networks. The initial deadline to reach the targeted number of at least 100 responses was set on September 30, 2023; however, as the target number of responses could not be reached, the deadline was extended 3 times until November 7, 2023.

### Demographics and Expertise Area of Respondents

A total of 115 survey respondents with expertise in the vaccine field in the European region completed the survey. Most respondents (n=91, 79.1%) stated that they answered the survey on their own behalf, while 24 (20.9%) respondents indicated that they responded on behalf of or were appointed by an organization. The distribution of countries where the institution, company, or organization of the survey respondent is based is presented in Table 1.

**Table 1.** The distribution of countries where the institution, company, or organization of the survey respondent is based.

Country	European region	Values, n (%)
Turkey	Southern Europe	29 (25.2)
Sweden	Northern Europe	16 (13.9)
Greece	Southern Europe	10 (8.7)
Spain	Southern Europe	8 (7)
The Netherlands	Western Europe	6 (5.2)
Austria	Western Europe	5 (4.3)
Norway	Northern Europe	5 (4.3)
Cyprus	Southern Europe	4 (3.5)
Germany	Western Europe	4 (3.5)
Switzerland	Western Europe	4 (3.5)
Australia	N/A^a^	3 (2.6)
Belgium	Western Europe	3 (2.6)
Italy	Southern Europe	3 (2.6)
Slovenia	Southern Europe	3 (2.6)
Denmark	Northern Europe	2 (1.7)
France	Western Europe	2 (1.7)
Lithuania	Northern Europe	2 (1.7)
Albania	Southern Europe	1 (0.9)
Canada	N/A	1 (0.9)
Ireland {Republic}	Northern Europe	1 (0.9)
Poland	Eastern or Central Europe	1 (0.9)
Singapore	N/A	1 (0.9)
United States	N/A	1 (0.9)

a NA: nonapplicable.

Table 2 shows the population groups that the respondents work toward improving the health of, and Table 3 exhibits the study areas or groups that are most relevant to the respondents’ areas of expertise.

**Table 2.** The distribution of the underrepresented groups that the survey respondents had experience with (N=115).

Groups^a^	Values, n (%)
All	38 (33)
Adults aged >65 y	51 (44.3)
Pregnant women	31 (27)
Lactating women	25 (21.7)
Children aged <5 y	35 (30.4)
Children aged 5‐11 y	25 (21.7)
Children aged 12‐17 y	28 (24.3)

a Experience among groups may be overlapping.

**Table 3.** Distribution of the area of expertise of the survey respondents.

Area of expertise	Values, n (%)
Civil Society (eg, NGOs^a^, patient advocacy groups, etc)	9 (7.8)
Funder (providers of nonrepayable funds such as public and other traditional R&D^b^ funders)	1 (0.9)
Industry	11 (9.6)
Policy and decision makers	7 (6.1)
Public health agency, including European (HERA^c^, ECDC^d^, EC^e^) and international (WHO^f^)	21 (18.3)
Regulatory Authority (including EMA^g^)	1 (0.9)
Scientific community (academia, RIs^h^ supporting vaccine R&D or other relevant global health fields)	73 (63.5)
Vaccine development alliances (biomedical R&D RIs [National, European, International, PDPs^i^, and other major initiatives])	5 (4.3)
I do not wish to specify	5 (4.3)

a NGO: nongovernment organizations.

b R&D: research and development.

c HERA: Health Emergency Preparedness and Response Authority.

d EDCD: European Centre for Disease Prevention and Control.

e EC: European Commission.

f WHO: World Health Organization.

g EMA: European Medicines Agency.

h RI: research infrastructure.

i PDP: product development partnership.

The expertise of the respondents was relatively equally divided among the 3 groups, whereas 1 in 3 respondents reported having experience with all groups. While 21.7% (25/115) of the respondents declared expertise at the entry and associate levels (<5 y) in the field of vaccines and vaccination, 39.1% (45/115) had expertise at the midsenior level (≥5 y), 27% (31/115) were at the director level, and 12.2% (14/115) were at executive levels. Overall, 44.3% (51/115) of the respondents had an experience of 15 years or more in the fields of vaccine and vaccination. Most (73/115, 63.5%) of the respondents were working in the scientific community, whereas 18.3% (21/115) and 9.6% (11/115) of them were working in public health agencies and the pharmaceutical industry, respectively. The geographical areas to which the respondents’ expertise is most related are presented in Table 4. There was a heterogeneous representation of the Southern Europe or Mediterranean region, followed by Western Europe, which constituted the most represented geographical regions.

**Table 4.** Distribution of the geographical area where the survey respondents’ expertise in vaccines is or are mostly related^a^.

Geographical area	Values, n (%)
Western Europe	39 (33.9)
Eastern Europe	17 (14.8)
Central Europe	11 (9.6)
Northern Europe	19 (16.5)
Southern Europe or Mediterranean	50 (43.5)

a Some respondents reported more than one geographical area.

### Reported Challenges in Recruiting Certain Population Groups in Vaccine Trials

#### Pediatric Population

**Figure 1.**
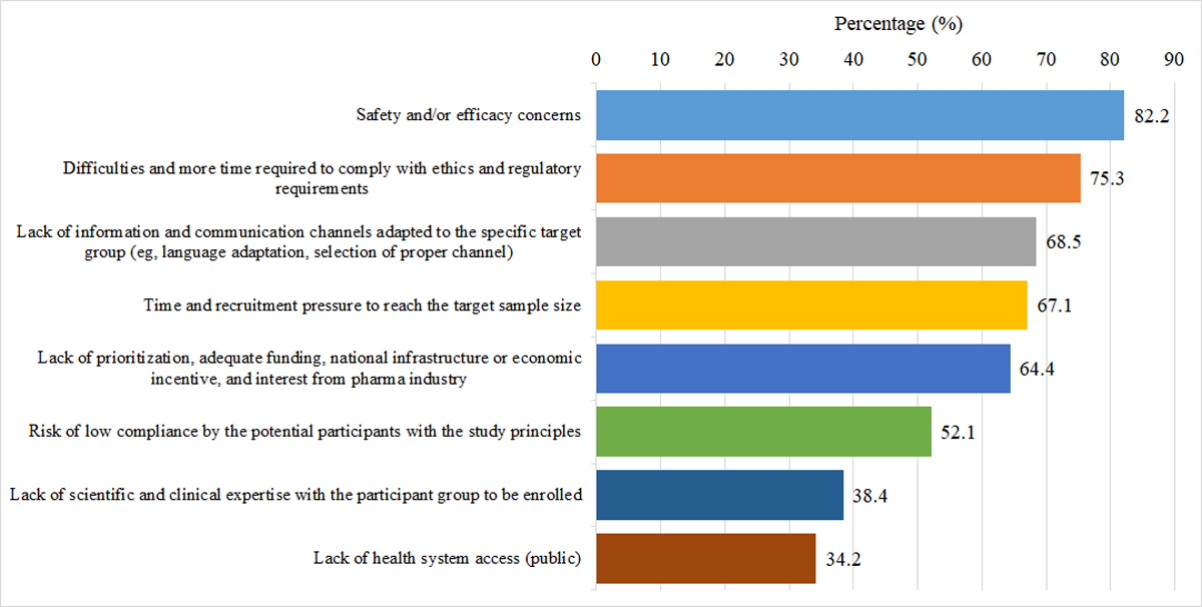
Distribution of reasons providers are unable to find or not willing to recruit a sufficient number of children participants in vaccine trials from the survey respondents’ perspective.

Figure 1 shows the challenges in recruiting children as participants; the 3 most pertinent being “safety and/or efficacy concerns” (60/73, 82.2%), “difficulties and more time required to comply with ethics and regulatory requirements” (55/73, 75.3%), and “lack of information and communication channels adapted to the specific target group” (50/73, 68.5%).

**Figure 2.**
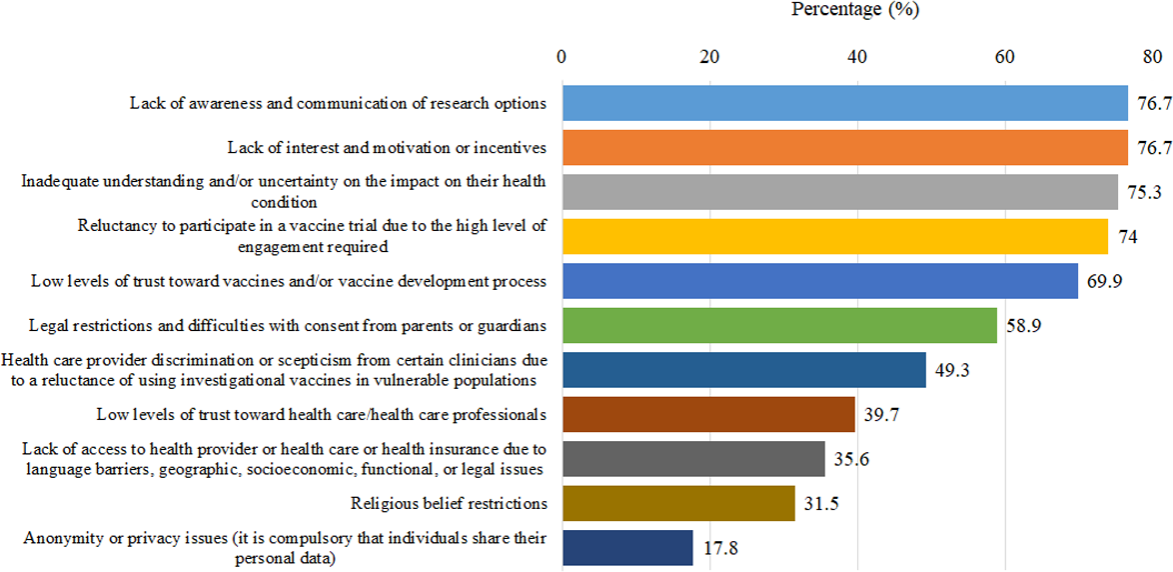
Distribution of the main reasons that children are not participating in vaccine trials from the survey respondents’ perspective.

The top 3 reasons why children are not participating in vaccine trials, as perceived by the responding professionals, were emphasized as “lack of awareness and communication of research options” and “lack of interest and motivation or incentives” (56/73, 76.7% for both), “inadequate understanding and/or uncertainty on the impact on their health condition” (55/73, 75.3%) and “reluctancy to participate in a vaccine trial due to the high level of engagement required” (54/73, 74%; Figure 2).

#### Pregnant and Lactating Women

**Figure 3.**
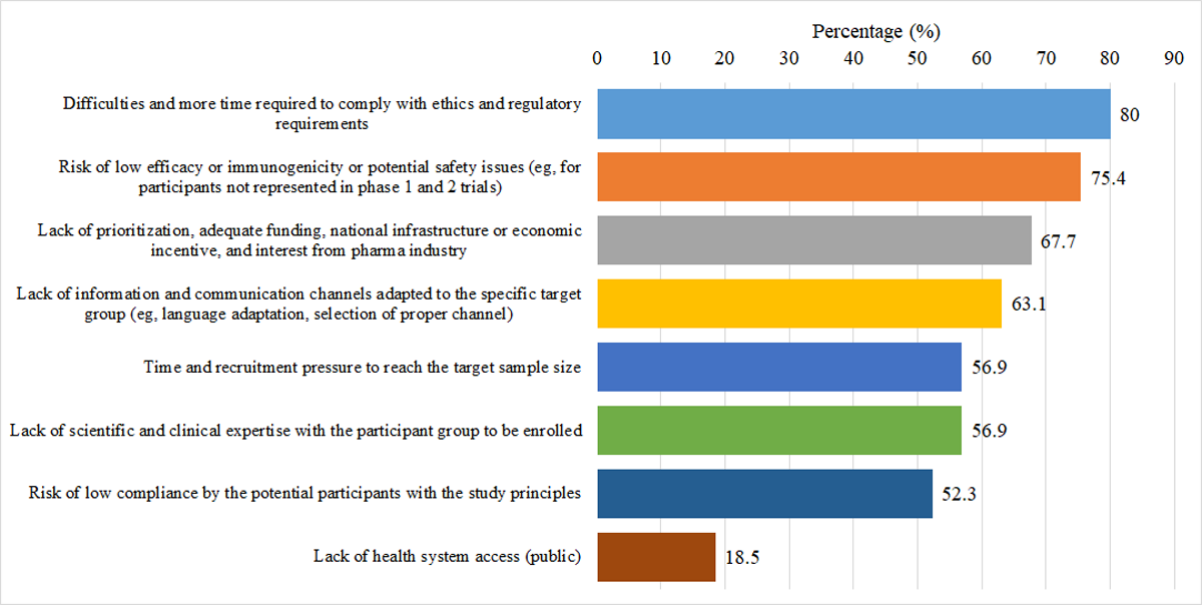
Distribution of reasons providers are unable to find or not willing to recruit a sufficient number of pregnant and lactating women participants in vaccine trials from the survey respondents’ perspective.

The main challenges in recruiting pregnant and lactating women in vaccine trials were “difficulties and additional time required to comply with ethics and regulatory requirements” (52/65, 80%), “risk of low efficacy or immunogenicity or potential safety issues (eg, for participants not represented in phase I and II trials)” (49/65, 75.4%), and “lack of prioritization, adequate funding, national infrastructure or economic incentive, and interest from pharma industry” (44/65, 67.7%; Figure 3).

**Figure 4.**
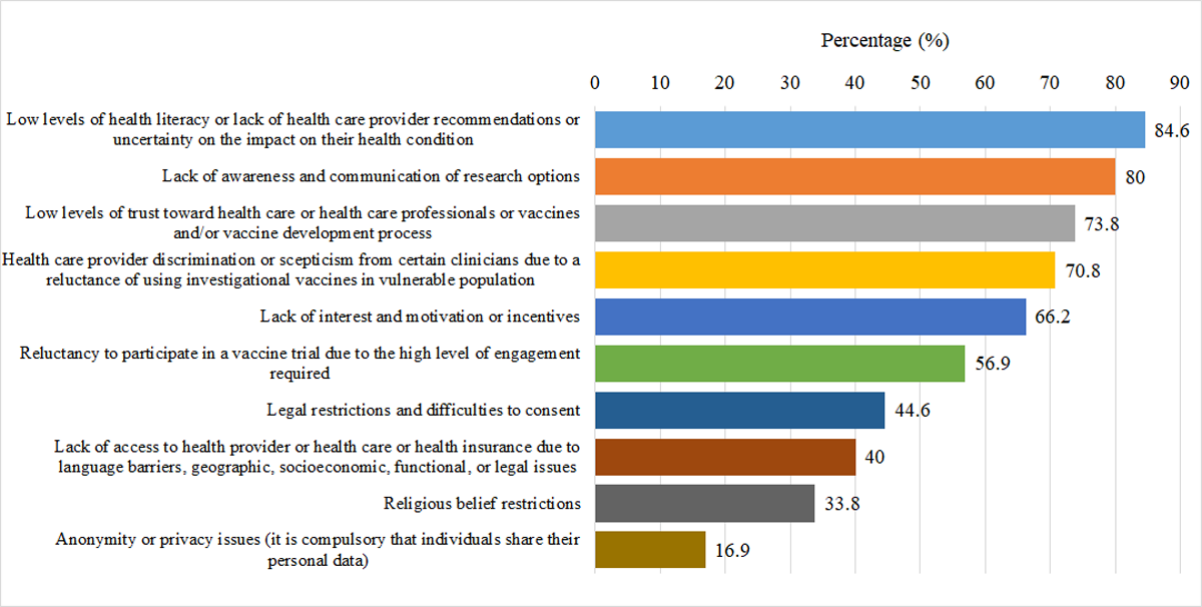
Distribution of the main reasons that pregnant and lactating women are not participating in vaccine trials from the survey respondents’ perspective.

“Low levels of health literacy or lack of health care provider recommendations or uncertainty on the impact on their health condition” (55/65, 84.6%), “lack of awareness and communication of research options” (52/65, 80%), and “low levels of trust toward health care, health care professionals, vaccines and/or vaccine development process” (48/65, 73.8%) were the main reasons why pregnant and lactating women are not participating in vaccine trials (Figure 4).

#### Older Population

**Figure 5.**
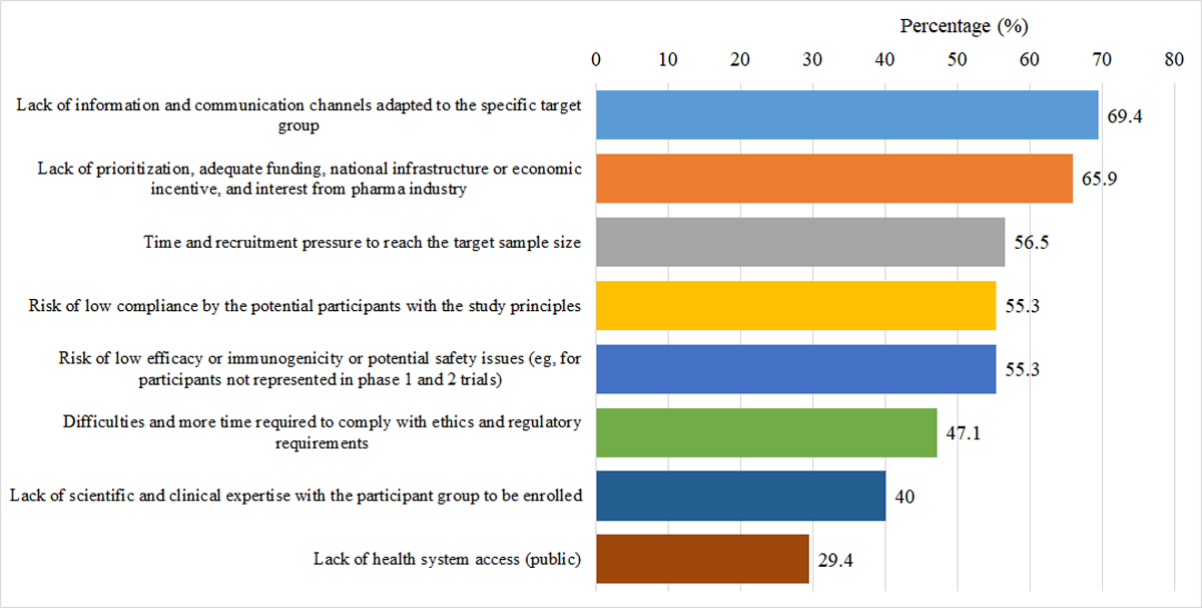
Distribution of reasons providers are unable to find or not willing to recruit a sufficient number of older adult participants in vaccine trials from the survey respondents’ perspective.

Figure 5 depicts the challenges in recruiting older adult participants. The 3 most frequent challenges were “lack of information and communication channels adapted to the specific target group” (59/85, 69.4%), “lack of prioritization, adequate funding, national infrastructure, or economic incentive and interest from pharma industry” (56/85, 65.9%), and “time and recruitment pressure to reach the target sample size” (48/85, 56.5%).

**Figure 6.**
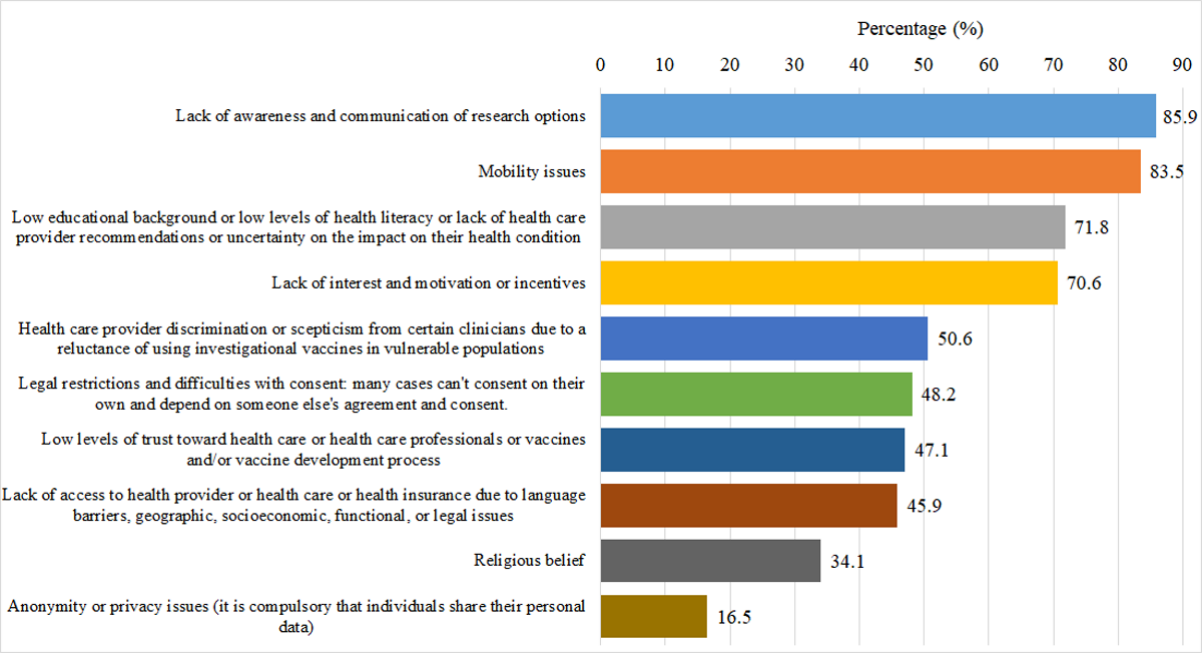
Distribution of the main reasons that older adults are not participating in vaccine trials from the survey respondents’ perspective.

The main barriers for which older adults are not participating in vaccine trials were “lack of awareness and communication of research options” (73/85, 85.9%), “mobility issues” (71/85, 83.5%), and “low educational background, low levels of health literacy, lack of health care provider recommendations or uncertainty on the impact on their health condition” (61/85, 71.8%; Figure 6).

#### Reported Facilitators in Recruiting Certain Population Groups in Vaccine Trials

The responses to the free-text question asking about how to improve equal access or improved representation in COVID-19 vaccine trials were mainly segregated into 5 categories: access and participation, collaboration and trust, information and communication, safety and compensation, and others. Factors that would motivate the participation in vaccine trials or studies are presented in Table 5.

**Table 5.** Distribution of the domains from the perspective of the survey respondents that motivate the participation of volunteers in a volunteer registry for vaccine trials or studies.

Motivation for participation	Values, n (%)
Provision of health-related incentives (including access to new treatments, getting expert medical care)	82 (71.3)
Provision of monetary incentives	59 (51.3)
Rapid access to disease-specific related information (eg, COVID-19 or Monkeypox)	64 (55.7)
Personal empowerment for active participation in the fight against an urgent public health threat	70 (60.9)
Sharing an opinion that influences the design of trials or studies	43 (37.4)

## Discussion

The recent COVID-19 pandemic demonstrated that new vaccine development against an emergent pathogen can prevent the loss of millions of people, restore the livelihoods of communities, and ensure safe travel. However, real-life evaluation of vaccine efficacy can be challenging due to the difficulties of recruiting different segments of the population and ensuring representation of the wider community with different sociodemographic characteristics. We have shown from the professionals’ perspective that recruitment of children, pregnant and lactating women, and older adults had specific challenges that required tailored approaches.

Unfortunately, there has been limited exploration of these challenges in vaccine trials from the perspective of other professionals to compare with the perspectives of our cohort. This is indeed one of the strengths of this study to provide novel findings where there is a knowledge gap. Studies have either explored the challenges based on race or ethnicity and primary language [20,21] or challenges in therapeutic trials such as cancer trials [22]. Therefore, we can only comment on the findings of other studies that report on the reflections of participants (either themselves or the caregivers of participants).

Recently, Meskell et al [23] published a Cochrane review on the factors that influence an individual’s choice to participate in a vaccine trial during a pandemic or epidemic. The trial team emerged as a significant factor, impacting how trial information was conveyed, the design of the trial for participant convenience, the provision of financial incentives, and access to additional support services. Personal considerations also played a role, including concerns regarding vaccine side effects, efficacy, and potential effects on daily life. Personal incentives encompassed access to vaccines, enhanced health outcomes, improved disease understanding, and the easing of pandemic or epidemic restrictions. Societal benefits included community assistance and contribution to scientific progress, often driven by memories of families and friends lost to the disease. Family and societal influences were also influential in potential participants’ decision-making processes. In another study, public information about COVID-19 vaccines and vaccine trials in Europe was compiled from official sources (eg, governments, public agencies, and nongovernment organizations). While information for the public was abundant across Europe, there was a large variation in number, type, and target audiences for the information among countries. Notably, little or no information was found for population groups that are typically underrepresented in vaccine clinical trials [12].

In this study, we surveyed professionals with expertise in vaccines or vaccinations to understand, for each underrepresented group, (1) the reasons why the providers are unable to find or unwilling to recruit participants from the underrepresented groups in vaccine trials and (2) the reasons why the individuals from the underrepresented groups are not participating in vaccine trials. The challenges in recruiting children and pregnant and lactating women were mainly shaped around “safety or efficacy concerns,” “difficulties about ethics and regulatory issues,” and “lack of targeted information and communication.” A rising threat of vaccine hesitancy among parents can be shaped around safety concerns. Caudal et al [24] illustrated that primary concerns revolved around vaccine adjuvants, the potential for short- and long-term adverse effects, and the risk of developing a disease or disability due to vaccination. Detoc et al [25] disclosed from the participant’s point of view that having a favorable opinion about vaccines was the only independent factor associated with agreement to enroll in a preventive vaccine trial. Acceptance of participation in vaccine trials was closely linked to a generally positive attitude toward vaccines. Notably, information provided by physicians was pivotal in the decision-making process for 70.2% of those who accepted participation. Similarly, parents whose children participated in the phase III clinical trial of the Oral Cholera Vaccine—Simplified disclosed logistical issues, costs, misinformation, and concerns about trial procedures and potential side effects as the major barriers [26]. Vaccine hesitancy poses a potential obstacle to recruitment in vaccine trials; therefore, enhancing altruism and ensuring the quality of information provided are crucial factors in fostering acceptance of participation in such trials.

Meanwhile, the reasons why children and pregnant and lactating women are not participating were mainly stated around “lack of awareness and communication,” “inadequate understanding and uncertainty,” and “trust issues.” Social capital, defined by Putnam, refers to “features of social organization such as networks, norms, and trust, that facilitate coordination for mutual benefit” [27]. Terraneo et al [28] found an association between social capital and the inclination to participate as volunteers in COVID-19 vaccine trials. These results may also be pertinent in encouraging parents and pregnant women to engage in vaccine trials. At the same time, fine-tuning the ethical and regulatory processes may not only accelerate recruitment but also help the sponsors to design trials that are not excluding these population groups right at the beginning. Interestingly, survey respondents stated “health care provider discrimination or skepticism due to a reluctance of using investigational vaccines” as a challenge in recruiting pregnant and lactating women, which corresponds to a lack of scientific and clinical knowledge not only among underrepresented populations but also among the health care providers. Upskilling the health care providers about vaccines and vaccinology or seeking researchers with expertise in pregnancy would be helpful to motivate the participation of pregnant and lactating women in vaccine trials.

Enrollment of older people has been a challenge during COVID-19 vaccine studies. However, it is crucial to ensure adequate representation of older individuals in clinical trials assessing preventive approaches to gain a comprehensive understanding of both the benefits and risks of interventions across the entire population. “Lack of information and communication channels adapted to the specific target group’’ was especially apparent as the main challenge in recruiting older adult participants. A French digital national platform, namely Covireivac, was set up in June 2020 [29]. An analysis of this registry underscored that computer-based recruitment methods might not be suitable for older individuals, despite the presence of a toll-free hotline and third-party registration options on the platform [30]. Conversely, in Germany, following public announcements regarding a planned phase I trial of the vaccine candidate MVA-SARS-2-S against SARS-CoV-2, volunteers successfully reached out to the study team via email. Remarkably, one-third of these volunteers were aged older than 60 years [31]. These data show that effective and culturally tailored combinations of communication strategies can increase the participation of volunteers among older age groups.

Among the main reasons why older adults are not participating in trials from the health care professionals’ perspective were also “lack of awareness and communication” and “low levels of health literacy” similar to the previous groups. However, a new challenge, “mobility issues,” was added as a challenge specific to this target group. Frailty among the older adults not only impacts mobility but also the ability to engage, and this may pose a significant barrier. Limited mobility and lack of social support for older adults may be one of the most important challenges in their participation in clinical trials but also the easiest challenge to overcome by careful planning and resource allocation. However, among a survey of 400 older adults living in Atlanta, Georgia, two-thirds of the participants admitted that they were willing to participate in vaccine clinical trials; however, one-third had had limitations due to physical, mental, or emotional problems [32]. The need to travel to the clinical research site was also salient as a barrier.

Provision of health-related incentives, including but not limited to access to new treatments and receiving expert medical care, seems to be the top-rated motivation for volunteers to participate in COVID-19 vaccine trials. However, it should also be considered that monetary incentives, in particular, might lead to an overrepresentation of financially disadvantaged groups [33].

Heterogeneous results with regard to the barriers for different underrepresented population groups demonstrate the need for tailored policies for each group. Andrasik et al [34] suggest that all clinical research sites are required to have active community advisory groups with clear lines of communication to clinical research site staff and leadership. To align all the stakeholders in vaccine development and implementation studies on the same agenda, the gap between the scientific community, public health community, and the policymakers should be closed. Hence, to convert the outputs of this task into societal impact, the results will be shared with diverse groups of stakeholders who play a role in vaccine studies. This can help to overcome some of the obstacles to enroll underrepresented population groups in clinical vaccine studies just by fine-tuning the processes such as ethics and regulatory processes.

The first limitation of the study was the narrower assessment of the underrepresented populations, focusing only on children, pregnant and lactating women, and older adults, while omitting other underrepresented groups (eg, socioeconomically disadvantaged populations, ethnic minorities, migrants, or individuals with disabilities). Another limitation was the fact that the extended survey was addressed to a very specific target population, that is, professionals with hands-on experience in vaccines and/or vaccinations. Given the lack of a systematic measurement of these professionals across countries, we could not perform representative sampling. Furthermore, we did not assess the perceptions of the patients or trial participants themselves. Future studies should elaborate on our findings and exhaustively explore means to reach results from more diverse and representative samples. A possible direction toward this goal is communication with national and international professional and expert organizations. Another limitation is that the survey covered responses from 23 countries, although Turkey, Sweden, and Greece (29, 16, and 10 respondents, respectively) constituted 47.8% of the represented countries. Most of the responses reflected the personal, not institutional, views of the respondents. Moreover, 81.7% (94/115) of the respondents were either from the scientific or public health community (73/115, 63.5% and 21/115, 18.3%, respectively), indicating the gap in the countries and the professional groups that could be reached. However, there was relatively good representation in terms of geographical regions and the population groups with which the survey respondents work, although the results cannot be generalized beyond Europe. Finally, we have used different response options instead of string responses for each underrepresented group in the survey for the sake of a quantitative descriptive analysis, but this might have been a potential source of bias as a few of the response options were not identical across groups. The responses should be interpreted as insights from experts in the vaccine field rather than reflections of trial participants. The results can therefore not directly point out the perspectives of participants from underrepresented populations but rather guide the design of future studies to get personal insights from these populations.

In conclusion, the major challenges stated by the providers for recruiting pregnant and lactating women and children were around safety or efficacy concerns and difficulties, and more time required to comply with ethics and regulatory requirements. On the other hand, the major challenges in recruiting participants from the older population were the lack of information and communication channels adapted to the specific target group and lack of prioritization, adequate funding, national infrastructure, or economic incentive and interest from pharma industry. While the lack of awareness and communication of research options was a common major perceived barrier for the participation of all the underrepresented groups, mobility issues emerged as another prominent factor for the older people. Lack of interest and motivation or incentives was another major perceived barrier for the children, while low levels of health literacy or lack of health care provider recommendations or uncertainty on the impact on their health condition were highlighted for the pregnant and lactating women. Improvement in communication infrastructure and channels to convey effective, easy-to-understand, and tailored messages to build trust in vaccines and convey the research options to motivate people to participate in vaccine trials seems to be a crucial step to increase the participation of the underrepresented groups.

## Supplementary material

10.2196/89025 Multimedia Appendix 1 VACCELERATE survey question. The questionnaire was developed specifically for this study.

10.2196/89025 Multimedia Appendix 2 VACCELERATE invitation letter.
