# Supplementary material for: Barriers and Opportunities to Include Underrepresented Population Groups in Vaccine Trials: Cross-Sectional, Observational, Online Survey Study From the VACCELERATE Research Network
Source: JMIR Public Health Surveill. 2026 Apr 7;12:e89025. doi: 10.2196/89025 (PMC13056233; doi:10.2196/89025)
Supplement: Multimedia Appendix 1 [file publichealth-v12-e89025-s001.pdf]

VACCELERATE Stakeholder Survey

Welcome

VACCELERATE (https://www.vaccelerate.eu) Expert Opinion/Stakeholder Survey

"Identifying barriers to participate in vaccine trials among pregnant and lactating women, children and older adults (over 65 years old)"

While the importance of representing diverse populations in vaccine trials is increasingly recognised, there are still significant improvements to be made regarding clinical trial inclusion. **VACCELERATE (https://www.vaccelerate.eu)** is a network of research institutes that envisions to streamline vaccine development for COVID-19 and for any future outbreaks, and to improve participation in vaccine trials across European countries.

Through this survey, we aim **to identify the barriers to participate in vaccine trials among pregnant and lactating women, children and adolescents, and older adults (over 65 years of age)**.

Secondary objectives include identifying:

- barriers against access to vaccine research
- barriers to vaccine trial enrollment of selected groups of individuals
- causes of underrepresentation in vaccine trials
- motivators in enrolling to vaccine trial volunteer registry

Who Is This Survey Addressed To?

If you are a professional with expertise or currently working in a field related to vaccine trials and vaccine development for pregnant and lactating women, and/or children and adolescents, and/or older adults (over 65 years of age), with full working proficiency of the English language, you are invited to participate.

The *survey* consists of 11 questions and will take approximately **10 minutes** to complete. Please read the questions thoroughly and answer as honestly as possible. There are no incorrect answers. You do not need to create an account. The questionnaire will be anonymous as no identifiable information will be collected and we will not be able to contact you.

Your participation is voluntary. You may withdraw from the survey at any time by simply closing your browser window. However, if you reach the end of the survey and click the submission button, your answers will be final and you will not be able to withdraw your submission. Your personal data will not be collected, and your submitted answers will be treated anonymously.

If you have any questions or concerns regarding the design or conduct of the survey, please contact us at muratt@omega-cro.com.tr.

This project has received funding from the European Union's Horizon 2020 research and innovation programme under grant agreement No. 101037867.

Who Are We?

VACCELERATE (https://www.vaccelerate.eu) is an EU-funded project (Grant Agreement no. 101037867) aiming to accelerate phase II & III COVID-19 vaccine clinical trials and help us better prepare for infectious disease outbreaks in the future.

The project is coordinated by the University Hospital of Cologne in Germany and is support by a large multidisciplinary group of expert from 23 different countries, including Austria, Belgium, Cyprus Czech Republic, Denmark, France, Germany, Greece, Hungary, Ireland, Israel, Italy, Lithuania, Netherlands, Norway, Poland, Portugal, Serbia, Slovakia, Spain, Sweden, Switzerland, and Turkey.

Protection of My Personal Data

Privacy and confidentiality are very important to us.

In the context of this survey data is collected and stored anonymously in our LimeSurvey and SPSS database to be used solely for research purposes. Therefore, your answers cannot be exploited and will not be shared with third parties, and will remain strictly confidential among partners in **VACCELERATE (https://www.vaccelerate.eu)** and will be stored on password-protected systems.

Your personal data is protected by the General Data Protection Regulation (EU) 2016/679 (including Art. 9 GDPR about processing of special categories of personal data) and the European Directive 2016/680 and National Legislation, for non-EU **VACCELERATE (https://www.vaccelerate.eu)** partner countries include Israel, Norway, Serbia, Switzerland, and Turkey. The law guarantees the right of access, correction, objection, processing and transferability of your data. In addition, it guarantees you the right to be notified should a breach of your data occur. For more information regarding the GDPR, you may visit **GDPR** (https://gdpr-info.eu/).

You may contact Prof. Murat Akova, M.D. at akova.murat@gmail.com for any further information or exercise of any right.

There are 20 questions in this survey.

In which capacity are you filling this survey out?

*

Please choose **only one** of the following:

- ◯ I am providing my personal views (expert opinion)
- ◯ I am representing an organisation / I have been appointed by the organisation

Which of the following population groups do you work towards improving the health of?

*

Please choose **all** that apply:

- ☐ Children under 5 years old
- ☐ Children 5-11 years old
- ☐ Children 12-17 years old
- ☐ Pregnant women
- ☐ Lactating women
- ☐ Adults over 65 years old
- ☐ All the above
- ☐ None of the above (If you selected this option, you are not eligible for filling in this questionnaire, and your participation will be terminated)

Which area of work/affiliation most closely relates to your expertise? (You can select more than one) *

Only answer this question if the following conditions are met:
Answer was 'All the above' *or* 'Adults over 65 years old' *or* 'Lactating women' *or* 'Pregnant women ' *or* 'Children 12-17 years old' *or* 'Children 5-11 years old' *or* 'Children under 5 years old' at question '2 [Q00002]' (Which of the following population groups do you work towards improving the health of?)

Please choose **all** that apply:

- ☐ Civil Society (e.g., non-government organisations (NGOs), patient advocacy groups, etc.)
- ☐ Funder (providers of non-repayable funds such as public and other traditional Research and Development (R&D) funders)
- ☐ Industry
- ☐ Policy and decision makers
- ☐ Public health agency, including European (HERA, ECDC, EC) and international (WHO)
- ☐ Regulatory Authority (including EMA)
- ☐ Scientific community (academia, Research Infrastructures (RIs) supporting vaccine R&D or other relevant global health fields)
- ☐ Vaccine development alliances (biomedical R&D RIs (National, European, International, PDPs and other major initiatives)
- ☐ I do not wish to specify

Which geographical area(s) is your expertise most related to?

*

Only answer this question if the following conditions are met:
Answer was 'All the above' *or* 'Adults over 65 years old' *or* 'Lactating women' *or* 'Pregnant women ' *or* 'Children 12-17 years old' *or* 'Children 5-11 years old' *or* 'Children under 5 years old' at question '2 [Q00002]' (Which of the following population groups do you work towards improving the health of?)

Please choose **all** that apply:

- ☐ Western Europe
- ☐ Eastern Europe
- ☐ Central Europe
- ☐ Northern Europe
- ☐ Southern Europe/Mediterranean

What is your highest level of expertise achieved in the organisation you are working/last worked for, in the field of vaccines and vaccination?

*

Only answer this question if the following conditions are met:
Answer was 'All the above' *or* 'Adults over 65 years old' *or* 'Lactating women' *or* 'Pregnant women ' *or* 'Children 12-17 years old' *or* 'Children 5-11 years old' *or* 'Children under 5 years old' at question '2 [Q00002]' (Which of the following population groups do you work towards improving the health of?)

Please choose **only one** of the following:

- ◯ Entry-level (0-2 years)
- ◯ Associate (2-5 years)
- ◯ Mid-senior level (5+ years)
- ◯ Director
- ◯ Executive

What is your total work experience in the fields of vaccines and vaccination? *

Only answer this question if the following conditions are met:
Answer was 'All the above' *or* 'Adults over 65 years old' *or* 'Lactating women' *or* 'Pregnant women ' *or* 'Children 12-17 years old' *or* 'Children 5-11 years old' *or* 'Children under 5 years old' at question '2 [Q00002]' (Which of the following population groups do you work towards improving the health of?)

Please choose **only one** of the following:

- ◯ 0-1 years
- ◯ 1-2 years
- ◯ 3-5 years
- ◯ 6-10 years
- ◯ 10-15 years
- ◯ 15 years or more

In your opinion, why are “providers” (i.e., those responsible for the trial designs and implementation) unable to find/not willing to recruit enough participants from children?

Please indicate the appropriate expressions for the following statements (Agree or Disagree)

*

Only answer this question if the following conditions are met:
Answer was 'All the above' *or* 'Children 12-17 years old' *or* 'Children 5-11 years old' *or* 'Children under 5 years old' at question '2 [Q00002]' (Which of the following population groups do you work towards improving the health of?)

Please choose the appropriate response for each item:

	Agree	Disagree
**Lack on information and communication channels adapted to the specific target group (e.g., language adaptation, selection of proper channel, etc.)**	○	○
**Lack of prioritisation, adequate funding, national infrastructure or economic incentive and interest from pharma industry**	○	○
**Time and recruitment pressure to reach the target sample size**	○	○
**Risk of low compliance by the potential participants with the study principles**	○	○
**Safety and/or efficacy concerns**	○	○
**Difficulties and more time required to comply with ethics & regulatory requirements**	○	○

	Agree	Disagree
**Lack of scientific and clinical expertise with the participant group to be enrolled**	◯	◯
**Lack of health system access (public)**	◯	◯

Here we would like to learn your opinions about the key barriers to accessing/participating in vaccine trials for children.

If you have any different opinion, please describe it.

Only answer this question if the following conditions are met:
Answer was 'All the above' *or* 'Children 12-17 years old' *or* 'Children 5-11 years old' *or* 'Children under 5 years old' at question '2 [Q00002]' (Which of the following population groups do you work towards improving the health of?)

Please write your answer here:

What do you think are the main reasons that children are not participating in trials? Please indicate the appropriate expressions for the following statements (Agree or Disagree) *

Only answer this question if the following conditions are met:
Answer was 'All the above' *or* 'Children 12-17 years old' *or* 'Children 5-11 years old' *or* 'Children under 5 years old' at question '2 [Q00002]' (Which of the following population groups do you work towards improving the health of?)

Please choose the appropriate response for each item:

	Agree	Disagree
**Lack of access to health provider/health care/health insurance – due to language barriers, geographic, socio-economic, functional, or legal issues**	○	○
**Lack of awareness and communication of research options**	○	○
**Lack of interest and motivation/incentives**	○	○
**Reluctancy to participate in a vaccine trial due to the high level of engagement required**	○	○
**Low levels of trust towards healthcare/healthcare professionals/**	○	○
**Low levels of trust towards vaccines and/or vaccine development process**	○	○
**Religious belief restrictions**	○	○

	Agree	Disagree
**Inadequate understanding and/or uncertainty on the impact on their health condition**	○	○
**Anonymity/Privacy issues (it is compulsory that individuals share their personal data)**	○	○
**Healthcare provider discrimination or scepticism from certain clinicians due to a reluctance of using investigational vaccines in vulnerable populations**	○	○
**Legal restrictions and difficulties with consent from parents/guardians**	○	○

If you have any different opinion, please describe it.

Only answer this question if the following conditions are met:
Answer was 'All the above' *or* 'Children 12-17 years old' *or* 'Children 5-11 years old' *or* 'Children under 5 years old' at question '2 [Q00002]' (Which of the following population groups do you work towards improving the health of?)

Please write your answer here:

In your opinion, why are "providers" (i.e., those responsible for the trial designs and implementation) unable to find/not willing to recruit enough participants from pregnant and lactating women? Please indicate the appropriate expressions for the following statements (Agree or Disagree) *

Only answer this question if the following conditions are met:
Answer was 'All the above' *or* 'Lactating women' *or* 'Pregnant women ' at question '2 [Q00002]' (Which of the following population groups do you work towards improving the health of?)

Please choose the appropriate response for each item:

	Agree	Disagree
**Lack on information and communication channels adapted to the specific target group (e.g., language adaptation, selection of proper channel, etc.)**	○	○
**Lack of prioritisation, adequate funding, national infrastructure or economic incentive and interest from pharma industry**	○	○
**Time and recruitment pressure to reach the target sample size**	○	○
**Risk of low compliance by the potential participants with the study principles**	○	○
**Risk of low efficacy/immunogenicity or potential safety issues (e.g. for participants not represented in phase 1 and 2 trials)**	○	○

	Agree	Disagree
**Difficulties and more time required to comply with ethics & regulatory requirements**	◯	◯
**Lack of scientific and clinical expertise with the participant group to be enrolled**	◯	◯
**Lack of health system access (public)**	◯	◯

Here we would like to learn your opinions about the key barriers to accessing/participating in vaccine trials for pregnant and lactating women.

If you have any different opinion, please describe it.

Only answer this question if the following conditions are met:
Answer was 'All the above' *or* 'Lactating women' *or* 'Pregnant women ' at question '2 [Q00002]' (Which of the following population groups do you work towards improving the health of?)

Please write your answer here:

What do you think are the main reasons that pregnant and lactating women are not participating in trials? Please indicate the appropriate expressions for the following statements (Agree or Disagree) *

Only answer this question if the following conditions are met:
Answer was 'All the above' *or* 'Lactating women' *or* 'Pregnant women ' at question '2 [Q00002]' (Which of the following population groups do you work towards improving the health of?)

Please choose the appropriate response for each item:

	Agree	Disagree
**Lack of access to health provider/health care/health insurance – due to language barriers, geographic, socio-economic, functional or legal issues**	○	○
**Lack of awareness and communication of research options**	○	○
**Lack of interest and motivation/incentives**	○	○
**Reluctancy to participate in a vaccine trial due to the high level of engagement required**	○	○
**Low levels of trust towards healthcare/healthcare professionals/vaccines and/or vaccine development process**	○	○
**Religious belief restrictions**	○	○

	Agree	Disagree
**Low levels of health literacy or lack of healthcare provider recommendations or uncertainty on the impact on their health condition**	○	○
**Anonymity/Privacy issues (it is compulsory that individuals share their personal data)**	○	○
**Healthcare provider discrimination or scepticism from certain clinicians due to a reluctance of using investigational vaccines in vulnerable populations**	○	○
**Legal restrictions and difficulties to consent**	○	○

If you have any different opinion, please describe it.

Only answer this question if the following conditions are met:
Answer was 'All the above' *or* 'Lactating women' *or* 'Pregnant women ' at question '2 [Q00002]' (Which of the following population groups do you work towards improving the health of?)

Please write your answer here:

In your opinion, why are "providers" (i.e., those responsible for the trial designs and implementation) unable to find/not willing to recruit enough older adult participants? Please indicate the appropriate expressions for the following statements (Agree or Disagree) *

Only answer this question if the following conditions are met:
Answer was 'All the above' *or* 'Adults over 65 years old' at question '2 [Q00002]' (Which of the following population groups do you work towards improving the health of?)

Please choose the appropriate response for each item:

	Agree	Disagree
**Lack on information and communication channels adapted to the specific target group (e.g. language adaptation, selection of proper channel, etc.)**	○	○
**Lack of prioritisation, adequate funding, national infrastructure or economic incentive and interest from pharma industry**	○	○
**Time and recruitment pressure to reach the target sample size**	○	○
**Risk of low compliance by the potential participants with the study principles**	○	○
**Risk of low efficacy/immunogenicity or potential safety issues (e.g., for participants not represented in phase 1 and 2 trials)**	○	○

	Agree	Disagree
**Difficulties and more time required to comply with ethics & regulatory requirements**	○	○
**Lack of scientific and clinical expertise with the participant group to be enrolled**	○	○
**Lack of health system access (public)**	○	○

Here we would like to learn your opinions about the key barries to accessing/participating in vaccine trials for older people (above 65 years of age).

If you have any different opinion, please describe it.

Only answer this question if the following conditions are met:
Answer was 'All the above' *or* 'Adults over 65 years old' at question '2 [Q00002]' (Which of the following population groups do you work towards improving the health of?)

Please write your answer here:

What do you think are the main reasons for older adults not participating in trials? Please indicate the appropriate expressions for the following statements (Agreed or Disagreed) *

Only answer this question if the following conditions are met:
Answer was 'All the above' *or* 'Adults over 65 years old' at question '2 [Q00002]' (Which of the following population groups do you work towards improving the health of?)

Please choose the appropriate response for each item:

	Agree	Disagree
**Lack of access to health provider/health care/health insurance – due to language barriers, geographic, socio-economic, functional or legal issues**	○	○
**Lack of awareness and communication of research options**	○	○
**Lack of interest and motivation/incentives**	○	○
**Mobility issues**	○	○
**Low levels of trust towards healthcare/healthcare professionals/vaccines and/or vaccine development process**	○	○
**Religious belief**	○	○
**Low educational background /low levels of health literacy/lack of healthcare provider recommendations or uncertainty on the impact on their health condition**	○	○

	Agree	Disagree
**Anonymity/Privacy issues (it is compulsory that individuals share their personal data)**	◯	◯
**Healthcare provider discrimination or scepticism from certain clinicians due to a reluctance of using investigational vaccines in vulnerable populations**	◯	◯
**Legal restrictions and difficulties with consent: many cases can't consent on their own and depend on someone else's agreement and consent.**	◯	◯

If you have any different opinion, please describe it.

Only answer this question if the following conditions are met:
Answer was 'All the above' *or* 'Adults over 65 years old' at question '2 [Q00002]' (Which of the following population groups do you work towards improving the health of?)

Please write your answer here:

What do you think would help you improve equal access/improved representation in COVID-19 vaccine research?

Only answer this question if the following conditions are met:
Answer was 'All the above' *or* 'Adults over 65 years old' *or* 'Lactating women' *or* 'Pregnant women ' *or* 'Children 12-17 years old' *or* 'Children 5-11 years old' *or* 'Children under 5 years old' at question '2 [Q00002]' (Which of the following population groups do you work towards improving the health of?)

Please write your answer here:

In your point of view, what would motivate individual's participation in a volunteer registry for vaccine trials / studies? *

Only answer this question if the following conditions are met:
Answer was 'All the above' *or* 'Adults over 65 years old' *or* 'Lactating women' *or* 'Pregnant women ' *or* 'Children 12-17 years old' *or* 'Children 5-11 years old' *or* 'Children under 5 years old' at question '2 [Q00002]' (Which of the following population groups do you work towards improving the health of?)

Please choose **all** that apply:

- [ ] Provision of health-related incentives (including access to new treatments, getting expert medical care)
- [ ] Provision of monetary incentives
- [ ] Rapid access to disease-specific related information (e.g., COVID-19 or Monkeypox)
- [ ] Personal empowerment for active participation in the fight against an urgent public health threat
- [ ] Sharing an opinion that influences the design of trials / studies

THANK YOU FOR PARTICIPATING AND COMPLETING OUR SURVEY

The record of your survey responses does not contain any identifying information about you, unless a specific

survey question explicitly asked for it.

If you used an identifying token to access this survey, please rest assured that this token will not be stored together with your responses. It is managed in a separate database and will only be updated to indicate whether you did (or did not) complete this survey. There is no way of matching identification tokens with survey responses.

Submit your survey.

Thank you for completing this survey.

LimeSurvey Appliance (https://www.turnkeylinux.org/limesurvey) - Powered by TurnKey Linux (https://www.turnkeylinux.org)
