# Supplementary material for: Barriers and Opportunities to Include Underrepresented Population Groups in Vaccine Trials: Cross-Sectional, Observational, Online Survey Study From the VACCELERATE Research Network
Source: JMIR Public Health Surveill. 2026 Apr 7;12:e89025. doi: 10.2196/89025 (PMC13056233; doi:10.2196/89025)
Supplement: Multimedia Appendix 2 [file publichealth-v12-e89025-s002.docx]

**Invitation Letter**

*Since we don’t collect identity information in the survey, we are unable to determine who has filled out the survey and who has not, therefore reminder messages are being sent to everyone.*

*If you have already filled out the VACCELERATE Survey, please ignore this message.*

*Thank you for your understanding.*

Dear Madam/Sir,

Help us to identify barriers in vaccine research for select populations, by completing our online survey.

The EU-funded research network VACCELERATE has created a survey targeted at **professionals** with **expertise in vaccines/ vaccinations**, with the aim to identify barriers to participation in vaccine trials among pregnant and lactating women, children and older adults (over 65 years old). The results of this survey are expected to help us more thoroughly understand what hampers the access to vaccine research and trial enrolment of the selected underserved groups.

If you are a professional in this field working with one or more these population groups, you are invited to participate in this survey. It consists of 11 questions and will take ~**10** minutes to complete. You can access the survey through this [link](https://lime.omega-cro.com.tr/index.php/422312?newtest=Y&lang=en). We would also be very happy if you could disseminate this survey to your network and pass on the link.

For any further information, feel free to contact the study leaders Dr. Murat Akova ([akova.murat@gmail.com](mailto:akova.murat@gmail.com)) and Dr. Dimitris Poulimeneas ([d.poulimeneas@cleoresearch.org](mailto:d.poulimeneas@cleoresearch.org)).

**Thank you for taking the time to participate in this survey**!

The VACCELERATE Team


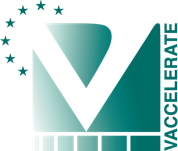

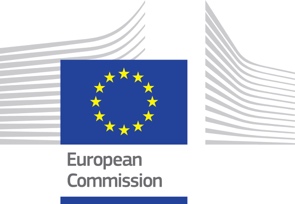

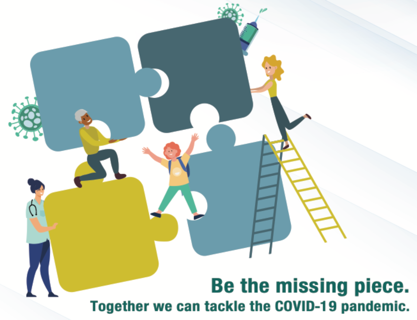


The pan-European project “VACCELERATE” is funded by the EC under Grant Agreement No 101037867. VACCELERATE is a network of research institutes that envisions to streamline vaccine development for COVID-19 and future outbreaks, and to improve participation in vaccine trials across European countries. More information: https://vaccelerate.eu/
